# Case Report: Anomalous Origin of the Right Coronary Artery From the Left Sinus of Valsalva With Aortic Dissection: New Myocardial Ischemia Mechanism

**DOI:** 10.3389/fcvm.2022.900803

**Published:** 2022-07-07

**Authors:** Zhongshang Xie, Junlin Zou, Hong Zhu, Haisong Bu

**Affiliations:** ^1^Department of Cardiovascular Surgery, Xiangya Hospital, Central South University, Changsha, China; ^2^National Clinical Research Center for Geriatric Disorders, Xiangya Hospital, Central South University, Changsha, China; ^3^Department of Cardiology, Xiangya Hospital, Central South University, Changsha, China

**Keywords:** the anomalous aortic origin of the right coronary artery, left sinus, aortic dissection, coronary blood flow, mechanism

## Abstract

The aortic anomaly of the right coronary artery (AAORCA) originating from the left aortic sinus (LCS) is a rare malformation that may result in sudden cardiac death (SCD), which may be due to the dilated aorta-pulmonary artery affecting the blood supply of the coronary artery. However, there are still some disputes about the treatment of the AAORCA. Herein, we present a rare case of AAORCA from the LCS with aortic dissection (AD). Considering the risk of dissection rupture and SCD, an emergency surgery of aortic replacement and coronary anomaly correction was performed successfully for the patient. This report illustrated that AAORCA complicated with acute AD (AAD) is lethal and may promote the occurrence of coronary ischemia or sudden death by a new “double-kill” mechanism that myocardial ischemia was based on the extent of a fixed and a dynamic component like slit-like ostium, proximal narrowing, acute take-off angle and intramural course with the elliptic vessel shape. There is no doubt that surgery is the best treatment option for the AAORCA with AAD.

## Introduction

The aortic anomaly of the right coronary artery (AAORCA) originating from the left aortic sinus (LCS) is a rare malformation, which has the possibility of sudden cardiac death (SCD) due to coronary artery ischemia. However, there are still some disputes about the treatment of the AAORCA (1, 2). Although most variants of AAORCA are benign, the challenge for clinicians is to recognize, which may attribute to the possibility of SCD, especially in the presence of strenuous exercise (3). Its mechanism has been attributed to the dilated aorta-pulmonary artery affecting the blood supply of the coronary artery (3, 4). Meanwhile, acute aortic dissection (AAD) is lethal and may promote the occurrence of SCD by a similar mechanism (5).

## Case Presentation

**Figure 1:**
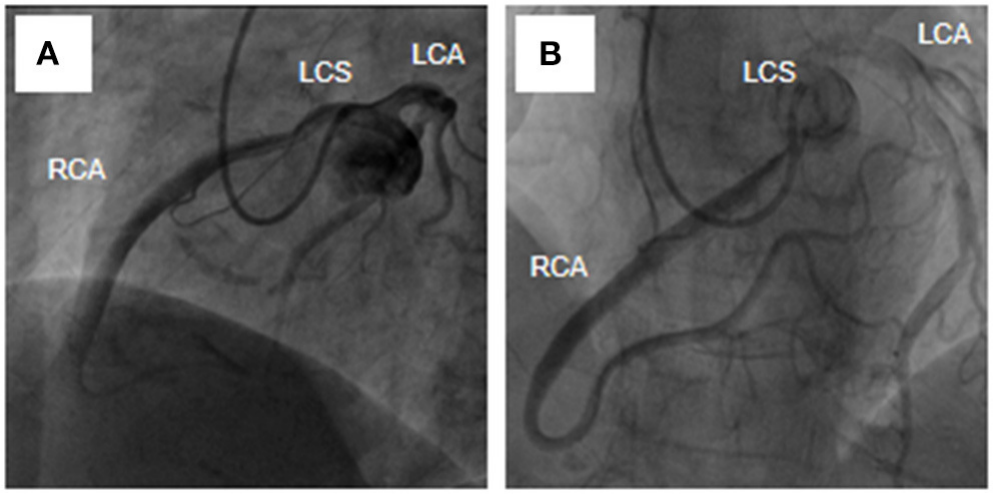
Transradia coronary angiography showed AAORCA **(A,B)**. AAORCA, the anomalous aortic origin of the right coronary artery; RCA, right coronary artery; LCS, left coronary sinus; LCA, left coronary artery.

**Figure 2:**
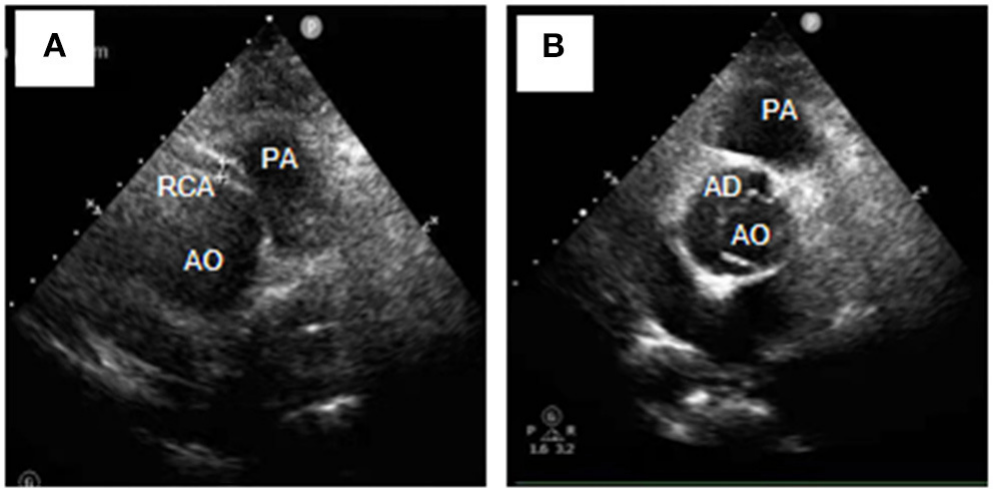
Transthoracic echocardiography (Short-axis view at the great artery) revealed Stanford type A aortic dissection **(A)**, avulsion of the RCA, and the RCA originating from the left coronary sinus **(B)**. RCA, right coronary artery; AD, aortic dissection; AO, aortic; PA, pulmonary artery.

**Figure 3:**
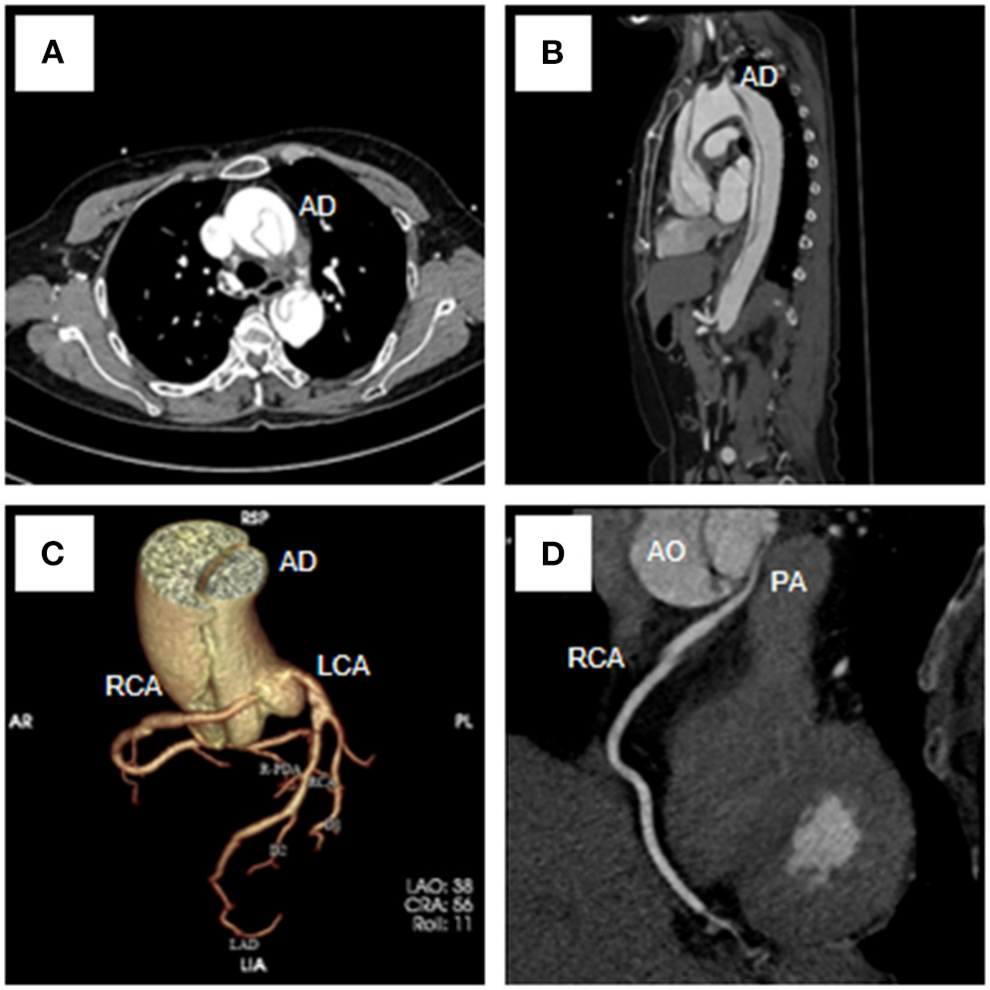
Total aorta plus coronary computed tomography angiogram showed the Stanford type A aortic dissection **(A–C)**, the RCA originated from the LCS **(C,D)** and coursed between the aorta and the pulmonary artery **(D)**. RCA, right coronary artery; LCA, left coronary artery; AD, aortic dissection; AO, aortic; PA, pulmonary artery.

A 54-year-old female was admitted urgently again because of sudden chest and back pain. At her first admission in June 2020, due to chronic atypical chest pain with paroxysmal syncope without any history, she was diagnosed with AAORCA and slight coronary stenosis (Figures 1A,B; Supplementary Video 1) after trans-radial coronary angiography and recovered after 2-week symptomatic treatment with medicine. At this time, no positive signs were found except a blood pressure of 162/90 mmHg at resting conditions and a slight diastolic murmur. An electrocardiogram (ECG) showed the mild ST-segment depression and cardiac troponin I was 0.17 ng/ml. Transthoracic echocardiography showed a Stanford type A aortic dissection (AD) with the widening from the aortic sinus to the thoracic aorta, avulsion injury at the opening of the right coronary artery (RCA), and the RCA originated from the left coronary sinus (LCS) (Figures 2A,B). Total aorta plus coronary CTA clarified the Stanford type A AD involving from the aortic root to the abdominal aorta, while left and right coronary arteries originated from the LCS (Figures 3A–C; Supplementary Video 2). The RCA was abnormally distributed and walked between the aorta-pulmonary artery (Figure 3D; Supplementary Video 3). Considering the risk of dissection rupture and SCD, an emergency surgery of aortic replacement and coronary anomaly correction was performed successfully for the patient. The AAORCA with AAD was identified and the aortic valves and LCS were still normal. We repaired the AAD with ascending aorta and total arch replacement combined with stent-graft elephant trunk technique, and corrected AAORCA with a right coronary artery bypass grafting. Postoperatively, the patient recovered well with medicine therapy including aspirin and clopidogrel, and was discharged on the 9th day after the operation. The patient is well now and has no limitations in daily activities at her 4-month follow-up visit.

## Discussion and Conclusions

The AAORCA is usually recognized when patients present with symptoms or are incidentally diagnosed, and its prevalence is between 0.008 and 0.32% of the population, which is considered to be due to differences in the definitions, imaging methods used and patient groups studied (3, 6). When RCA originates from the contralateral sinus and abnormally distributes between the aorta-pulmonary artery, there is the possibility of sudden cardiac death, which may be due to the dilated artery affecting the blood supply of the coronary artery (3, 4). However, the interarterial course between the aorta-pulmonary artery may only represents a surrogate for ischemia-associated anatomical high-risk features like slit-like ostium, acute take-off angle, proximal narrowing (also referred to as hypoplasia) with elliptic vessel shape and intramural course, which referred to a two-tier concept for the pathomechanisms of ischemia in AAORCA. In this concept, the occurrence of ischemia is based on the extent of a fixed (anatomic high-risk features of slit-like ostium and proximal narrowing) and a dynamic (acute take-off angle, intramural course with the elliptic vessel shape) component (7). In our case, the patient's first hospitalization in June 2020 may be due to myocardial ischemia caused by a fixed component like proximal narrowing similar to classic coronary lesions. However, the patient with AAORCA suffered from an AAD in this emergency admission. AAD is a life-threatening disease that demands urgent surgery. The proximal aortic lesion of the AAD not only increased the risk of rupture but also increased the risk of myocardial infarction. About 2% of all AAD patients have an acute myocardial infarction (AMI), which may be attributed to the exogenous compression of the enlarged pseudolumen on the opening of the coronary artery or the obstruction of the intimal valve (4). Meanwhile, the AAD increased the risk of myocardial infarction in patients with AAORCA by slit-like ostium, proximal narrowing, acute take-off angle and intramural course with the elliptic vessel shape.

Although echocardiographic diagnosis has been described to diagnose AAORCA (6), cardiac catheterization and angiocardiography are still considered the gold criterion diagnostic strategy. Recently, coronary CTA has gradually been used to differentiate and diagnose AAORCA. CTA is a non-invasive diagnostic aid, which can accurately show the origin and distribution of the coronary artery, evaluate and describe its anatomical relationship with surrounding structures (8). Hence, the non-invasive detection method is widely considered and selected to describe the anatomical structure of the coronary artery.

All patients with AAORCA, especially combined with AAD, should be treated with emergency surgery. The dilated aorta compressed the RCA located between the aorta-pulmonary artery and was considered to be the cause of AMI. The enlargement of the sinus of Valsalva in AD also possibly led to acute myocardial infarction. Therefore, patients with AAORCA from the contralateral sinus of Valsalva complicated with AAD should be intervened early, especially in high-risk patients (hypertension or Marfan syndrome) with AAD (3). Meanwhile, AAD with multiple arterial malformations may be a new syndrome caused by a certain gene or chromosome similar to Down syndrome or Marfan syndrome (9). Moreover, the coronary anomaly may be a risk for aortic dissection with possible hemodynamic abnormalities in the aortic root and increase the difficulty of aortic root replacement (10). However, for AAD patients with coronary artery abnormalities, we recommend the use of coronary CTA for clear diagnosis to avoid the risk of dissection rupture. Taken together, this report illustrated that patients with AAORCA from the contralateral sinus of Valsalva complicated with AAD may result in AMI by the new “double-kill” mechanism that myocardial ischemia was based on the extent of a fixed and a dynamic component like slit-like ostium, proximal narrowing, acute take-off angle and intramural course with the elliptic vessel shape.

## Data Availability Statement

The original contributions presented in the study are included in the article/Supplementary Material, further inquiries can be directed to the corresponding author/s.

## Ethics Statement

The studies involving human participants were reviewed and approved by the Ethics Committee of Xiangya Hospital of Central South University, Changsha, China. The patients/participants provided their written informed consent to participate in this study. Written informed consent was obtained from the individual(s) for the publication of any potentially identifiable images or data included in this article.

## Author Contributions

ZX, HZ, and HB prepared and wrote the main manuscript. JZ, HZ, and HB prepared figures and supplementary videos. HB and ZX collected, checked, and analyzed the data. All authors have read and approved the final manuscript and agreed to be accountable for the content of the work.

## Funding

This work was supported by the Natural Science Foundation of Hunan (2022JJ40765) and the Natural Science Foundation of Changsha City, China (kq2202366).

## Conflict of Interest

The authors declare that the research was conducted in the absence of any commercial or financial relationships that could be construed as a potential conflict of interest.

## Publisher's Note

All claims expressed in this article are solely those of the authors and do not necessarily represent those of their affiliated organizations, or those of the publisher, the editors and the reviewers. Any product that may be evaluated in this article, or claim that may be made by its manufacturer, is not guaranteed or endorsed by the publisher.

## Supplementary Material

Supplementary Video 1 Transradia coronary angiography showed AAORCA.

Supplementary Video 2 The computed tomography angiogram showed the RCA originated from the LCS.

Supplementary Video 3 The computed tomography angiogram showed the RCA abnormally distributed and walked between the aorta-pulmonary artery.
