# Supplementary material for: Case Report: Anomalous Origin of the Right Coronary Artery From the Left Sinus of Valsalva With Aortic Dissection: New Myocardial Ischemia Mechanism
Source: Front Cardiovasc Med. 2022 Jul 7;9:900803. doi: 10.3389/fcvm.2022.900803 (PMC9300863; doi:10.3389/fcvm.2022.900803)
Supplement: Supplementary file 4 [file Data_Sheet_1.PDF]

CARE case report guidelines

CARE Checklist of information to include when writing a case report

(cc) BY-NC-ND

Topic	Item	Checklist item description	Reported on Line
Title	1	The diagnosis or intervention of primary focus followed by the words "case report"	line 1, page 1
Key Words	2	2 to 5 key words that identify diagnoses or interventions in this case report, including "case report"	line 36, page 2
Abstract (no references)	3a	Introduction: What is unique about this case and what does it add to the scientific literature?	line 24, page 2
	3b	Main symptoms and/or important clinical findings	line 28, page 2
	3c	The main diagnoses, therapeutic interventions, and outcomes	line 28, page 2
	3d	Conclusion—What is the main "take-away" lesson(s) from this case?	line 31, page 2
Introduction	4	One or two paragraphs summarizing why this case is unique (**may include references**)	line 45, page 3
Patient Information	5a	De-identified patient specific information.	line 56, page 3
	5b	Primary concerns and symptoms of the patient.	line 56, page 3
	5c	Medical, family, and psycho-social history including relevant genetic information	line 58, page 3
	5d	Relevant past interventions with outcomes	line 57, page 3
Clinical Findings	6	Describe significant physical examination (PE) and important clinical findings.	line 61, page 3
Timeline	7	Historical and current information from this episode of care organized as a timeline	line 59, page 3
Diagnostic Assessment	8a	Diagnostic testing (such as PE, laboratory testing, imaging, surveys).	line 59, page 3
	8b	Diagnostic challenges (such as access to testing, financial, or cultural)	line 62, page 3
	8c	Diagnosis (including other diagnoses considered)	line 62, page 3
	8d	Prognosis (such as staging in oncology) where applicable	line 60, page 3
Therapeutic Intervention	9a	Types of therapeutic intervention (such as pharmacologic, surgical, preventive, self-care)	line 68, page 4
	9b	Administration of therapeutic intervention (such as dosage, strength, duration)	line 68, page 4
	9c	Changes in therapeutic intervention (with rationale)	line 68, page 4
Follow-up and Outcomes	10a	Clinician and patient-assessed outcomes (if available)	line 69, page 4
	10b	Important follow-up diagnostic and other test results	line 69, page 4
	10c	Intervention adherence and tolerability (How was this assessed?)	not applicable
	10d	Adverse and unanticipated events	not applicable
Discussion	11a	A scientific discussion of the strengths AND limitations associated with this case report	line 71, page 4
	11b	Discussion of the relevant medical literature **with references**.	line 71, page 4
	11c	The scientific rationale for any conclusions (including assessment of possible causes)	line 101, page 5
	11d	The primary "take-away" lessons of this case report (without references) in a one paragraph conclusion	line 101, page 5
Patient Perspective	12	The patient should share their perspective in one to two paragraphs on the treatment(s) they received	line 89, page 5
Informed Consent	13	Did the patient give informed consent? Please provide if requested	Yes ☑ No ☐
